# Pyroptosis: A Developing Foreland of Ovarian Cancer Treatment

**DOI:** 10.3389/fonc.2022.828303

**Published:** 2022-02-07

**Authors:** Tianyi Liu, Min Hou, Manyu Li, Cheng Qiu, Lin Cheng, Tianyu Zhu, Jinfeng Qu, Lanyu Li

**Affiliations:** ^1^ Department of Medical Oncology, National Cancer Center/National Clinical Research Center for Cancer/Cancer Hospital, Chinese Academy of Medical Sciences and Peking Union Medical College, Beijing, China; ^2^ Center for Reproductive Medicine, Cheeloo College of Medicine, Shandong University, Jinan, China; ^3^ Key Laboratory of Reproductive Endocrinology of Ministry of Education, Shandong University, Jinan, China; ^4^ Shandong Key Laboratory of Reproductive Medicine, Jinan, China; ^5^ Shandong Provincial Clinical Research Center for Reproductive Health, Jinan, China; ^6^ National Research Center for Assisted Reproductive Technology and Reproductive Genetics, Shandong University, Jinan, China; ^7^ Cheeloo College of Medicine, Shandong University, Jinan, China; ^8^ Department of Orthopaedic Surgery, Qilu Hospital, Cheeloo College of Medicine, Shandong University, Jinan, China; ^9^ Department of Obstetrics and Gynecology, Central Hospital Affiliated to Shandong First Medical University, Jinan, China

**Keywords:** pyroptosis, ovarian cancer, gasdermin, inflammasome, caspase, cell death

## Abstract

Ovarian cancer (OVCA) has the second highest mortality among all gynecological cancers worldwide due to its complexity and difficulty in early-stage diagnosis and a lack of targeted therapy. Modern strategies of OVCA treatment involve debulking surgery combined with chemotherapy. Nonetheless, the current treatment is far from satisfactory sometimes and therefore the demand for novel therapeutic measures needs to be settled. Pyroptosis is a notable form of programmed cell death characterized by influx of sodium with water, swelling of cells, and finally osmotic lysis, which is distinctive from numerous classes of programmed cell death. So far, four major pathways underlying mechanisms of pyroptosis have been identified and pyroptosis is indicated to be connected with a variety of disorders including cancerous diseases. Interestingly enough, pyroptosis plays an important role in ovarian cancer with regard to long non-coding RNAs and several regulatory molecules, as is shown by previously published reports. In this review, we summarized major pathways of pyroptosis and the current research foundations of pyroptosis and ovarian cancer, anticipating enriching the thoughts for the treatment of ovarian cancer. What is more, some problems yet unsolved in this field were also raised to hopefully propose several potential threads of OVCA treatment and research directions in future.

## Introduction

Among all gynecological cancers, ovarian cancer (OVCA) does not represent the largest portion of new cases, but it is the cancer type with the second highest mortality worldwide (1, 2). Although the incidence has almost been stable for several years, OVCA is still estimated as the fifth cancer death reason for American women in 2021 due to its complexity and difficulty in early-stage diagnosis and a lack of targeted therapy (3). Moreover, the ovarian cancer patients usually show no evident symptoms at the early stage. Even in advanced OVCA patients, some certain symptoms including back pain, fatigue, abdominal pain, bloating, constipation, and urinary symptoms cannot guarantee an accurate diagnosis, nor can the exploratory laparotomy (4, 5). Based on histopathological characteristics, ovarian cancers can be divided into three main types including epithelial, germ cell, and sex-cord-stromal types (6, 7). Surgery is undoubtedly the foundation of treating ovarian cancer. However, it is far from satisfactory and the traditional treatment of advanced ovarian cancer has become the combination of surgery and chemotherapy (7–9). Accordingly, many novel drugs selectively acting on specific targets such as prexasertib specifically inhibiting cell cycle checkpoint kinase (Chk) 1/2 have been developed for certain classifications of OVCA (10). Nevertheless, prexasertib acting as a Chk 1/2 inhibitor is now under investigation for the treatment of high-grade serous OVCA, whereas its promising efficacy has been preliminarily evidenced only in phase 1 studies on account of its moderate hematological toxicity (11). Therefore, larger confirmatory studies are required to evaluate these new drugs and innovative methods of treating other types of OVCA are needed as well.

Programmed cell death (PCD) is an essential biological process in all multicellular organisms, underlying many physiological progressions involving growth and development, anti-infection, and survival in extreme condition (12, 13), etc. Moreover, diseases comprising neoplasm, autoimmune diseases, infection, etc., could emerge when PCD is interrupted. Several famous forms of PCD have been well acknowledged so far, encompassing apoptosis, autophagy, necroptosis, ferroptosis, and pyroptosis (14). Apoptosis is characterized by cytoplasmic shrinkage, nuclear condensation, and the maintenance of completeness of membranes and organelles. Many molecules are involved in apoptosis, and the key initiators are caspase-2, -8, -9, and -10 while the main executioners are caspase-3, -6, and -7 (13, 15, 16). Autophagy is distinguished by the formation of autophagosomes, with the indispensable autophagy-related proteins. Moreover, caspase-2, -3, -6 and -8 are found to work as regulators (16–18). Necroptosis, a programmed cell death similar to necrosis, is realized by the activation of receptor-interacting protein kinase 3 (RIPK3)-mixed lineage kinase domain-like pseudokinase (MLKL) pathway and the downregulation of caspase-8 simultaneously (14). As another newfound PCD, the physiological roles of ferroptosis remain intangible but it shows great potential in tumors. Therefore, it is a promising area of cancer treatment (18, 19).

More recently, pyroptosis, an inflammatory PCD, is made up of two Greek roots “pyro” and 'ptosis', which is presumed to happen in response to infection and is reported to be triggered by inflammasomes customarily. After the discovery of pyroptosis in the field of infection, the scope of research was gradually extended and pyroptosis has been revealed to be of vital importance in many other diseases, including metabolic diseases (20), cardiovascular diseases (21), neurological diseases (22). As inflammation is evidently one of the hallmarks of cancers (23), a strong association might exist between pyroptosis and malignant diseases. Importantly, in recent years, some chemotherapeutic agents have been found to stimulate the formation of inflammasomes, hinting that there may be a correlation between cancer treatment and pyroptosis (24, 25). Generally speaking, with activation of caspase-1, -4 (in human), -5 (in human), and -11 (in mice) and cleavage of gasdermins (GSDMs), plasma membrane pores subsequently form as a result of N-termini of GSDMs and cause membrane perforation, cell swelling, plasma membrane lysis, chromatin fragmentation, and release of intracellular proinflammatory contents, which distinguishes pyroptosis from apoptosis biochemically and morphologically (14, 17, 26, 27). Moreover, great strides have been made in detecting the underlying mechanisms of pyroptosis, broadening our understanding of cancers and providing new threads of cancer management.

Hereof, in this review, we mainly summarized some cardinal mechanisms of pyroptosis and discussed the relationship between pyroptosis and ovarian cancer with an emphasis on the current study foundations, hopefully to provide some potential perspectives in OVCA treatment.

## Main Mechanisms of Pyroptosis: Setting the Cells on Fire

### The Gasdermin Family

The gasdermin family is a cluster of proteins encoded by GSDM family genes, including GSDMA, GSDMB, GSDMC, GSDMD, GSDME, and PJVK. All the members share a similar structure containing a C-terminal repressor domain (RD) and an N-terminal pore-forming domain (PFD). Besides, there exists a linker region in all GSDMs except for PJVK. Significantly, the N-terminus and C-terminus are highly conserved in the GSDM family, while the linker regions are diverse (28), resulting in cleavage by different caspases or granzymes. Once the cleavage occurs, RD and PFD fall apart, and hence PFD could come into play. Then the PFD binds to membrane phospholipids and generates pores (29). The GSDM family possesses extensive functions and is widely expressed in human, although regrettably, a lot of detailed mechanisms are still unknown. Moreover, pyroptosis, as yet, is proved to be associated with GSDMB, GSDMD, and GSDME (30). GSDMA, related to mitochondrial homeostasis (31) and an increased apoptosis-inducing activity in human mucus-secreting pit cells, is found to be inhibited in gastric cancers (32). The biological functions of GSDMC and PJVK remain unknown, but it is reported that the expression level of GSDMC is positively correlated with the metastatic ability of melanoma cells (33), indicating the possible relationship between GSDMC and tumorigenesis.

### The Canonical Pathway

**Figure 1:**
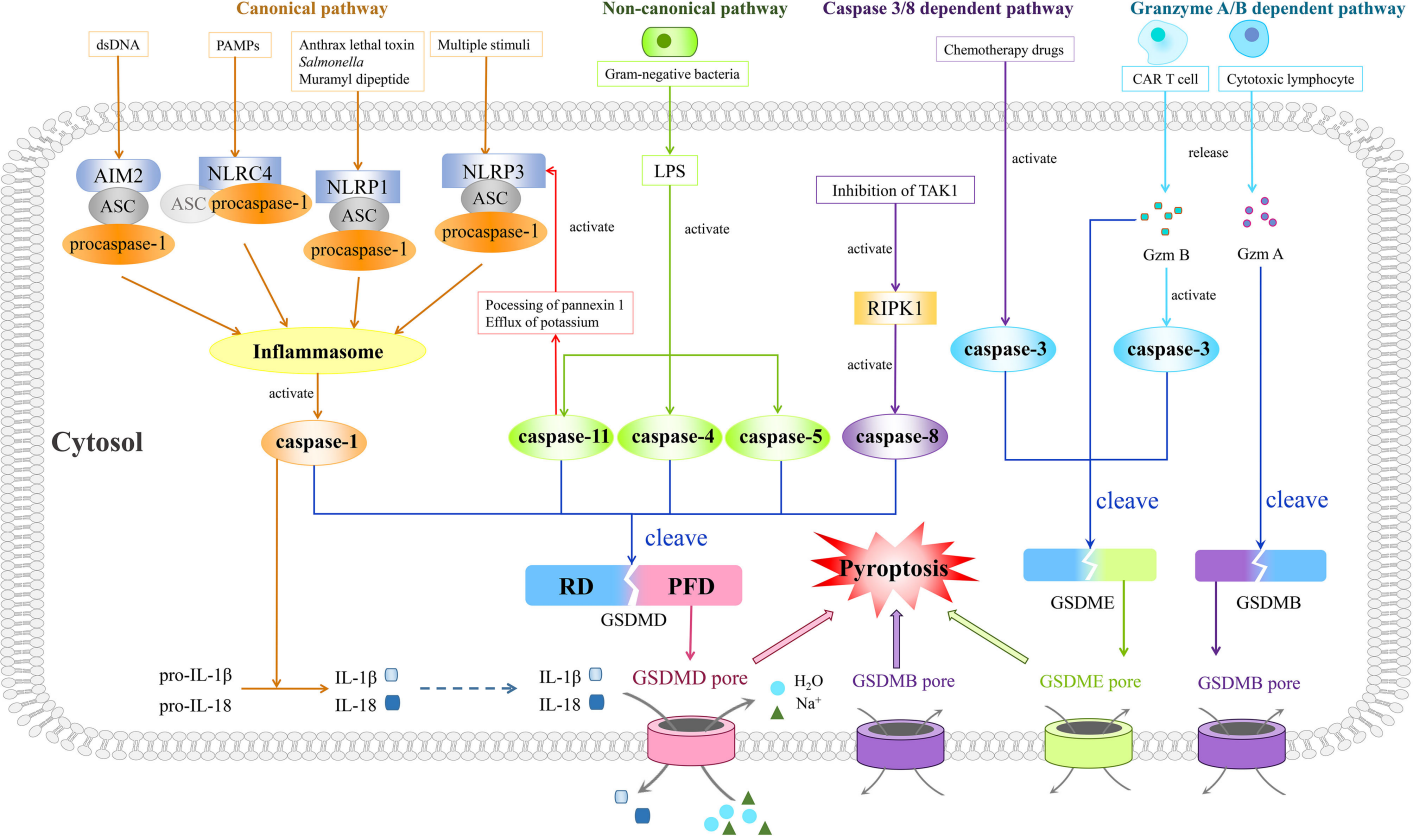
Four prestigious pathways indicated in mechanisms of pyroptosis. Of note is that the canonical pathway is composed of inflammasomes, caspase-1, and GSDMD. Moreover, the inflammasome complex consists of PRRs (NLRP1, NLRP3, NLRC4, and AIM2), procaspase-1, and ASC, with the last one being dispensable in NLRC4 inflammasome. Different PRRs constitute corresponding types of inflammasomes and recognize different types of PAMPs or DAMPs. After recognition of PAMPs or DAMPs, the assembled inflammasomes activate caspase-1, thus cleaving GSDMD. The gasdermin pore formed by N-terminus of GSDMD results in pyroptosis characterized by outlet for IL-1β and IL-18, influx of sodium with water, swelling of cells, and finally osmotic lysis. In the non-canonical pathway, LPS derived from gram-negative bacteria could trigger pyroptosis through activating caspase-4, -5, and -11 to cleave GSDMD. Besides, the activated caspase-11 could also inspire the activation of the NLRP3 inflammasome. As for the caspase 3/8-dependent pathway, activated RIPK1 by inhibition of TAK1 helps caspase-8 to cut GSDMD and to mediate pyroptosis while the activated caspase-3 by chemotherapeutic drugs could split GSDME, leading to pyroptosis as well. In the granzyme A/B-dependent pathway, Gzm B released by CAR T cells could induce GSDME-modulated pyroptosis by both direct cleavage of GSDME and indirect cleavage of GSDME *via* activation of caspase-3, while cytotoxic lymphocyte-released Gzm A cleaves GSDMB to induce pyroptosis.

As pyroptosis was first coined in 2001, it is mostly concerned with inflammation (34) and largely depends on the assembly of a crucial component, the inflammasome complex, which is composed of pattern-recognition receptors (PRRs), procaspase-1, and apoptosis-associated speck-like protein containing a caspase recruitment domain (ASC) (**Figure 1**). The activation of canonical inflammasomes mostly appears in macrophages and dendritic cells (35).

PRRs of canonical inflammasomes often cover NLRP1, NLRP3, and NLRC4, absent in melanoma 2 (AIM2), with these four proteins constituting four corresponding types of inflammasomes. The first three belong to the nucleotide-binding oligomerization domain (NOD)-like receptor (NLR) family, with NLRP possessing a pyrin domain (PYD) and NLRC possessing an N-terminal caspase recruitment domain (CARD) (36). AIM2 is endowed with a PYD and a DNA-binding HIN-200 domain (37), and the latter decides the connection between AIM2 and endogenous or pathogen-derived DNA (38). PYD and CARD of these inflammasome receptors contribute to recognition of certain pathogen-associated molecular patterns (PAMPs) and damaged-associated molecular patterns (DAMPs) (36, 39). For example, the NLRP1 inflammasome mediates the recognition of lethal toxin from *Bacillus anthracis*, muramyl dipeptide, and *Salmonella* (40–42), whereas the NLRP3 inflammasome recognizes multiple stimuli, including PAMPs such as Sendai virus, influenza, and bacterial pore-forming toxins, as well as DAMPs such as extracellular ATP, hyaluronan, and glucose (35, 43–47). Additionally, the NLRC4 inflammasome recognizes PAMPs including flagellin and muramyl dipeptide (48, 49), while the AIM2 inflammasome only recognizes endogenous or pathogen-derived double-stranded DNA (dsDNA) (38).

PAMPs and DAMPs are activated to recruit inflammasome adaptors ASC after recognition by PRRs. PYD and CARD are contained in ASC as well, similar to that of PRRs and participating in a homotypic interaction. The PYD–PYD interaction helps PRRs to summon ASC, and in the meantime CARD of ASC is indispensable for recruiting procaspase-1 into the inflammasome complex *via* CARD–CARD interaction (50). Apart from recruiting procaspase-1, ASC is indispensable in the maturation of IL-1β (51). Besides, NLRP1B and NLRC4 probably recruit procaspase-1 directly as they have CARD themselves (52). Moreover, the self-cleavage of procaspase-1 could give rise to caspase-1 activation primarily in macrophages and dendritic cells (53–55) (**Figure 1**).

Caspase-1, also referred to as interleukin-1-beta-converting enzyme, is another pivotal core in this pathway, distinguishing pyroptosis from apoptosis (56). It was first described as an inflammatory cysteine protease by Thornberry et al. in 1995 (57). After being recruited to inflammasomes, the concentration of regional caspase-1 monomers increases and consequently the dimerization might be accelerated (58), since the dimeric form of caspase-1 has protease activity. In caspase-1, there exists a CARD domain linker between the CARD domain and C-terminus, along with an interdomain linker inside the C-terminus which separates it into a larger subunit (p20) and a smaller one (p10) (59). As these two linkers could be self-cleaved by caspase-1 at diverse sites (60), the p20 subunit and p10 subunit are separated to reunite the active tetramer which is composed of two p20 subunits and two p10 subunits (61). Also, following research revealed that active caspase-1 could transform precursors of IL-1β and IL-18 into mature forms (62), while cleaving GSDMD into two termini as well (53). Then, the N-terminus of GSDMD, PFD, could generate a gasdermin pore in the plasma membrane when the inhibitory RD is cleaved apart. These pores bring about the outlet for IL-1β and IL-18, the influx of sodium with water, the swelling of cells, and finally the osmotic lysis (29, 63–65) (**Figure 1**). Intriguingly, in gastric cancer cells, the expression of GSDMD is downregulated according to a previously published article, which results in abnormal proliferation of cancer cells (66), indicating that elevating the expression of GSDMD might inhibit the progression of gastric cancer.

### Non-Canonical Pathway

Unlike that of the canonical pathway, the non-canonical pathway requires caspase-4 and -5 in human and ortholog caspase-11 in mice (67, 68). In the 1990s, the study by Li found that caspase-1 knockout mice showed high resistance to the injection of lipopolysaccharide (LPS) (69). Moreover, following articles described possible mechanisms. It was found that caspase-11 is expressed in a great quantity due to the stimulation of LPS (70). This expression causes the induction of pyroptosis in macrophages, which possibly depends on the ATP-mediated P2X7 signaling pathway according to Yang et al. They observed the instantly fast release of extracellular ATP after transfection of LPS in bone marrow-derived macrophages, mediated by the cleavage of pannexin-1 depending on caspase-11 (71). ATP finally triggered the activation of P2X7, leading to its opening with ion movement, formation of larger pores on the membrane, and following pyroptosis (72, 73). Besides, the stimulation of LPS results in potassium’s efflux, in which pannexin-1 is indispensable. Caspase-11 somehow could activate NLRP3 inflammasome mentioned in the canonical pathway, for the efflux of potassium plays a critical part in this procession (67, 71, 74). The direct combination of LPS and orthologs of caspase-11, caspase-4, and caspase-5 could induce the activation of caspases themselves (68, 75, 76). All these activated caspases engender the cleavage of GSDMD resembling that of caspase-1 and ensuing pyroptosis as mentioned above (53, 77, 78) (**Figure 1**). In a study conducted by Yokoyama et al., it was revealed that secretoglobin 3A2 was capable of inhibiting growth of human non-small cell lung cancer (NSCLC) and colorectal cancer (CRC) cells in the mouse metastasis model by means of the caspase-4-mediated non-canonical pyroptosis pathway (79).

According to a study analyzing the caspase-1, -4, and -5 gene mutations in cancers, it is indicated that inhibition of caspase-5 probably contributes to carcinogenesis in microsatellite instability-positive tumor entities (80). Terlizzi et al. also found that in patients with NSCLC, the circulating level of caspase-4 is raised compared with those without (81). With further diligent work, their recent study clearly declared that caspase-4 is highly expressed in NSCLC compared to normal lung tissues, while caspase-11 motivates the development of lung cancer in mice. Notably, this high expression of caspase-4 is associated with a poor survival rate in NSCLC patients (82).

### Caspase 3/8-Dependent Pathway

In 2017, Feng and colleagues firstly demonstrated the novel function of caspase-3 in pyroptosis, breaking the stereotype that pyroptosis could be induced only by inflammatory caspases. In their experiment, chemotherapy drugs could mediate the caspase-3-governed cleavage of GSDME, exposing its gasdermin N-terminal domain and executing pyroptosis as well (**Figure 1**). Moreover, TNF-induced apoptosis was also found to be switched to pyroptosis by GSDME1 (83). Their results were later reconfirmed in various sorts of cancers, including gastric cancer (84), lung cancer (85), and colon cancer (86). Besides, in murine macrophages, it was indicated that when the traditional canonical NLRP3-inflammasome pathway is blocked, its activators like ATP could induce pyroptosis through the caspase-3/GSDME pathway, a switch between apoptosis and pyroptosis in cancers (87), instead of the caspase-1/GSDMD pathway (88). Briefly, the switch between pyroptosis and apoptosis is primarily determined by the expression level of GSDME, and both the PCD pathways are caspase-dependent. When GSDME is highly expressed, active caspase-3 cleaves it in two termini with the N-terminal domains punching holes on the cell membrane and causing pyroptosis. Conversely, apoptosis will occur if there is a low expression level of GSDME. However, more studies are needed to reconfirm the mechanisms underlying this switch (87).

Only 1 year later in 2018, two back-to-back studies revealed that inhibition of TGF-β-activated kinase-1 (TAK1) by *Yersinia* YopJ has the ability to provoke pyroptotic cell death in murine macrophages during *Yersinia* infection (89, 90). They uniformly agreed that during the aforementioned process, TAK1 blockade by *Yersinia* bacteria could lead to activation of RIPK1, together with the subsequent activation of caspase-8, and caspase-8 could chop GSDMD, finally unleashing IL-1β as a result of the pores formed by N-termini of GSDMDs (89, 90) (**Figure 1**). This process was then reassured by Schwarzer et al. in intestinal epithelial cells in a gut inflammation model (91). Moreover, intriguingly, in two recent works, caspase-8 was regarded as the pivot of the apoptosis–necroptosis–pyroptosis network (92, 93), exhibiting its shining role in cell death.

### Granzyme A/B-Dependent Pathway

So far, five subtypes of human granzymes (Gzms) have been described in natural killer cells and cytotoxic T lymphocytes whereas eleven subtypes of murine granzymes are now known to us (94). Among all, Gzm A and B are of vital importance, which also function in cell death, inflammation, infection, and tumor immunity (95). Over the years, much attention has been given to Gzm A and B in cell death, where their roles in either caspase-dependent or caspase-independent cell death are well explained. Moreover, perforin, a 67-kDa protein guarding the entrance of granzymes, is widely expressed in immune cells and could induce cell apoptosis in synergy with granzymes (96).

In January of last year, Liu et al. described their conclusion that chimeric antigen receptor (CAR) T cells stimulate caspase-3 to cut GSDME through unleashing granzyme B, the function of which is to cleave and activate caspase-3 in cooperation with perforin, and thus pyroptosis happens in target cells (97). Shortly afterward, Zhang et al. reported that Gzm B could split GSDME without the existence of caspase-3. In other words, Gzm B could induce GSDME-modulated target tumor cell pyroptosis by both direct cleavage of GSDME and indirect cleavage of GSDME *via* activation of caspase-3 (98) (**Figure 1**). Additionally in the same year, it was demonstrated that other than Gzm B, Gzm A also takes effect as a pyroptosis executioner. In GSDMB-positive cells, natural killer cells and cytotoxic T lymphocytes cause cell death through pyroptosis. What is more, cytotoxic lymphocytes are confirmed to release Gzm A, which then specifically cuts GSDMB through the interdomain with the help of perforin as well, resulting in pyroptosis (**Figure 1**). Furthermore, this remarkable pathway could successfully promote tumor clearance in mice (99), providing a new paradigm for pyroptosis and cancer treatment.

## Current Research Foundations of Pyroptosis and Ovarian Cancer

### Genes That Might Regulate Pyroptosis in OVCA

With more studies focusing on pyroptosis and ovarian cancer, it was not so long ago that Berkel et al. published their paper comparing differential expression and copy number variations of certain GSDM family members in normal ovarian tissues with those of malignant serous ovarian tissues (100). They firstly pointed out that the expression of GSDME is downregulated whereas GSDMD and GSDMC are expressed at a high level in serous OVCA, which is associated with a poor prognosis of *TP53*-mutated OVCA patients. Likewise, as executioners of GSDMs, the expression of caspase-1, -3, -4, -5, and -8 is decreased at the mRNA level in serous ovarian cancer. Also, the copy number variation events happen more frequently in genes encoding GSDMD and GSDMC, in accordance with their expression. Additionally, various histological subtypes of epithelial ovarian cancer express GSDMB and GSDME differently (100) **(** **Table 1** **)**.

**Table 1:** Current research foundations of pyroptosis and ovarian cancer.

Year	Authors	Research object	Ovarian cancer cell lines	Gate molecules	Signaling pathways	Additional information
2021	Berkel et al. (100)	Differential expression and copy number variations of GSDMs	/	/	/	In epithelial ovarian cancer, the expression of GSDMB is increased in mucinous histotype compared to endometrioid and serous histotypes. Also, the expression of GSDMD is elevated in clear cell and serous histotypes compared to endometrioid histotype.
2021	Ye et al. (101)	Pyroptosis-related genes	/	/	/	The 13 downregulated genes include PRKACA, GSDMB, SCAF11, PJVK, CASP9, NOD1, PLCG1, NLRP1, GSDME, ELANE, TIRAP, CASP4, and GSDMD while the 18 upregulated genes are GPX4, NLRP7, NLRP2, CASP3, CASP6, TNF, IL1B, IL18, CASP8, NLRP6, GSDMA, GSDMC, PYCARD, CASP5, AIM2, NOD2, NLRC4, and NLRP3.
2018	Li et al. (102)	LncRNA GAS5	SKOV3, OVCAR-3, A2780, and 3AO	GSDMD	The canonical pathway	Depletion of lncRNA GAS5 promotes viability of OVCA cells, while the overexpression of lncRNA GAS5 inhibits proliferation and colony formation in OVCA cells.
2021	Tan et al. (103)	LncRNA HOTTIP	CAOV-3, A2780, SKOV3, and OVCAR3	GSDMD	ASK1/JNK signaling pathway	LncRNA HOTTIP is upregulated in ovarian cancer tissues, and microRNA-148a-3p was a downstream target gene of HOTTIP, exerting negative effects on the regulatory functions of HOTTIP.
2020	Liang et al. (104)	Osthole	A2780 and OVCAR3	GSDME	/	Osthole could mediate GSDME-dependent pyroptosis while suppressing cell death by mitochondria-mediated apoptosis and causing cell autophagy in OVCA.
2020	Zhang et al. (105)	Nobiletin	A2780 and OVCAR3	GSDMD, GSDME	/	Nobiletin could inhibit cell proliferation, induce apoptosis *via* DNA damage in a dose-dependent way, and mediate pyroptosis through induction of autophagy in OVCA cells.
2019	Qiao et al. (106)	α-NETA	Ho8910, Ho8910PM, Hey, SKOV3, and A2780	GSDMD	GSDMD/caspase-4 pathway	α-NETA treatment causes epithelial ovarian cancer cell membrane blistering and cytoplasm leakage, typical manifestations of cells undergoing pyroptosis, which could be arrested by β-arrestin-2.

Secondly yet importantly, not long ago Qi and colleagues identified 31 differentially expressed genes (DEGs) that might regulate pyroptosis between OVCA and normal ovarian tissues, based on which the OVCA cases were classified. Among the 31 DEGs, 13 genes were downregulated while the remaining 18 genes were enriched in the tumor tissues. Moreover, a total of 7 DEGs including 3 downregulated (PLCG1, ELANE, and PJVK) and 4 upregulated (AIM2, CASP3, CASP6, and GSDMA) genes were retained for generating a prognostic model and a risk model because of their significant *p*-values, where 3 genes (PLCG1, ELANE, and GSDMA) were shown to be risk factors, while the other 4 genes (AIM2, PJVK, CASP3, and CASP6) were protective in the TCGA cohort. Thereafter, prognostic value was evaluated and pyroptosis-related genes were ascertained to play a key role in tumor immunity and predicting the prognosis of OVCA (101) **(** **Table 1** **)**.

### LncRNAs and Pyroptosis in OVCA

**Figure 2:**
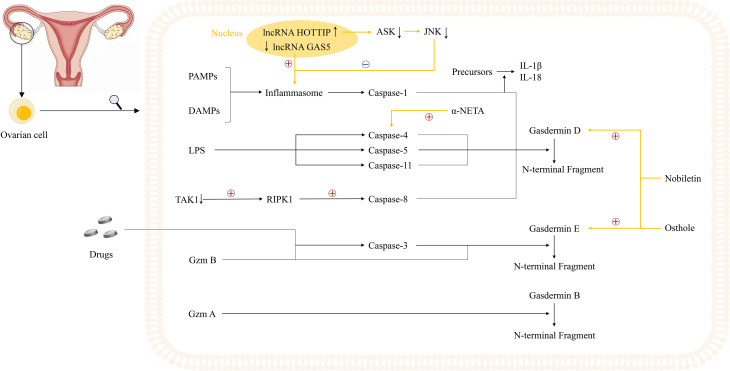
Potential mechanisms underlying pyroptosis in ovarian cancer cells and current study foundations. Notably, two lncRNAs, GAS5 and HOTTIP, play an important role in the regulation of inflammasomes. The inhibited expression of lncRNA GAS5 in ovarian cancer could trigger the formation of inflammasome while lncRNA HOTTIP is highly expressed in ovarian cancer, the knockdown of which leads to upregulation of ASK1/JNK signaling, elevated formation of NLRP1-inflammasome, and pyroptosis. Moreover, three novel small molecules including osthole, nobiletin, and α-NETA are reported to regulate the pyroptosis process in ovarian cancer cells. Osthole and nobiletin are of high similarity since they both have an effect on ROS production, MMP, and LC3-related autophagy. However, osthole could mediate GSDME-dependent pyroptosis while nobiletin could mediate pyroptosis through GSDMD- and GSDME-dependent ways. Moreover, α-NETA treatment causes epithelial ovarian cancer cell death through pyroptosis, with dramatically augmented level of GSDMD and caspase-4.

Alternatively, two studies revealed that two long non-coding RNAs (lncRNAs), lncRNA growth arrest-specific transcript 5 (GAS5) and lncRNA HOXA transcript at the distal tip (HOTTIP), could regulate the pyroptosis process in OVCA, serving as a good cop and a bad cop, respectively (102, 103). Li et al. determined the positive effect of lncRNA GAS5 on pyroptosis in OVCA. Not only did they determine the repressed expression of lncRNA GAS5 in ovarian cancer tissues, but also they used lncRNA GAS5 overexpression and depletion models to identify that lncRNA GAS5 triggers the formation of inflammasome, thus leading to pyroptosis both *in vivo* and *in vitro* (102). The work done by Tan et al. was more complicated, with several downstream effectors discovered. In ovarian cancer tissues and cell lines, lncRNA HOTTIP is upregulated, the knockdown of which could lead to pyroptosis, hampering the progression of OVCA. Mechanistically, silencing lncRNA HOTTIP brings about upregulation of its downstream target gene microRNA (miRNA)-148a-3p, low AKT2 expression, positive modulation of the ASK1/JNK signaling pathway, and elevated formation of NLRP1-inflammasome (103) (**Figure 2**, **Table 1** **)**. In view of the broad research prospects of pyroptosis in OVCA, more potential lncRNAs that could modulate pyroptosis are yet to be unearthed.

### Several Regulatory Molecules of Pyroptosis in OVCA

Meanwhile, some reports showed that apart from lncRNAs, pyroptosis in OVCA could also be induced by various molecules comprising osthole, nobiletin, and 2-(alpha-naphthoyl)ethyltrimethylammonium iodide (α-NETA) (104–106). Osthole, a natural compound found in several medicinal plants such as *Cnidium monnieri* and *Angelica pubescens*, is reported to show potential anticancer, antioxidant, antimicrobial, and anti-inflammatory activities (107, 108). Similarly, nobiletin is another plant-derived natural compound targeting various oncogene and onco-suppressor pathways, thus showing great anticancer activity (109, 110). Moreover, α-NETA is a stable, non-competitive, slowly reversible choline acetylcholine transferase inhibitor (106).

Recently, Liang et al. have found that osthole could mediate GSDME-dependent pyroptosis while eliciting reactive oxygen species (ROS) generation, decreasing mitochondrial membrane potential (MMP), and inducing LC3-mediated autophagy. In their study, the level of cleavage of GSDME was raised by osthole, exerting tremendous influence on the occurrence of pyroptosis (104). Remarkably, and perhaps not coincidentally, Zhang et al. uncovered the new identity of nobiletin as the pyroptosis trigger in OVCA in the same year. Highly similar to osthole, nobiletin could also stimulate ROS production, decrease MMP, and promote the evocation of classical autophagy in connection with LC3. Besides, nobiletin was verified to evoke the pyroptosis process in an autophagy-related, ROS-mediated, GSDMD- and GSDME-dependent way, slightly different from that of osthole (105). What is more, a later published paper further convinced that α-NETA treatment causes epithelial ovarian cancer cell death through pyroptosis, with a dramatically augmented level of GSDMD, caspase-4, LC3B, and IL-18 secretion (106) **(** **Figure 2** **,** **Table 1** **)**.

### Characteristics of Pyroptosis in OVCA and Other Types of Cancer Cells

The pyroptosis process happens not only in OVCA cells but also in many other types of tumor cells. For example, in NSCLC patients, GSDMD is highly expressed, the same as that in malignant serous ovarian tissues, and indicates a poor prognosis as well. Moreover, in digestive system carcinomas, caspase-1 is demonstrated to be low-expressed in hepatocellular carcinoma and colorectal cancer (111). Surprisingly in colorectal cancer, lncRNA RP1-85F18.6 is reported to promote proliferation and invasion as well as suppress pyroptosis (112) whereas lncRNA nuclear paraspeckle assembly transcript 1 (NEAT1) could mediate ionizing radiation-induced pyroptosis relying on upregulation of GSDME expression (113). Besides, as a platinum antitumor agent, lobaplatin could remarkably elevate the level of ROS in CRC cells and phosphorylate JNK. Then activated JNK could cause mitochondrial damage and release of cytochrome C, promoting caspase-3 and -9 cleavage and GSDME-dependent pyroptosis, which shows a moderate overlap between OVCA and CRC (86).

## Discussion

Taken together, as a notable style of lately identified programmed cell death, pyroptosis displays a significant role in multitudinous diseases embodying cancerous ailments (84, 86, 104), infectious diseases (90, 114), neurological diseases (115, 116) and cardiovascular events (117, 118). Among them, nevertheless, carcinomas are emerging as one of the auspicious prospects. Moreover, as is conspicuously stated above, compelling evidence denotes a close relation between pyroptosis and ovarian cancer. With four major pathways of pyroptosis being discovered one after another, the gasdermin family becomes the kernel of pyroptosis induction, and caspases that have the capacity to mediate pyroptosis are no longer confined to inflammatory ones. Therefore, questions are gradually starting to surface. Are the existing pathways complete mechanisms of pyroptosis? We now know that caspases triggering pyroptosis, for example caspase-3 and -8, could also participate in apoptosis. Particularly, caspase-8 serves as hub of the apoptosis–necroptosis–pyroptosis network, whose bigger potential needs to be tapped. So is there a chance that other apoptosis-related caspases, such as caspase-2, -6, -7, -9, and -10, could also function in pyroptosis? For this reason, a grand network involving apoptosis-related caspases and yet undetected further GSDMs is worth looking forward to.

What is more, since mounting studies demonstrated an association between pyroptosis and tumor immunotherapy, it might be possible to treat cancer patients with immunotherapy assisted by pyroptosis-inducing nanoparticles (119–121) in the future. It was reported that one of those nanoparticles could mediate tumor cell pyroptosis in a mouse colon carcinoma model, and the pyroptotic tumor cells could release DAMPs, thus initiating adaptive immunity and boosting the efficacy of immune checkpoint inhibitors (ICIs) (120). However, the safety of those nanoparticles should be taken into consideration when applied. Additionally, it was also reported that ICIs could kill resistant tumors only in the context of the concomitant induction of pyroptosis (122), highlighting the importance of the combination of pyroptosis inducers and ICIs in treating ICI-resistant tumors. Nevertheless, since the occurrence of pyroptosis brings about the release of inflammatory components, which might promote the development of tumors (123, 124), pyroptosis, as a double-edged sword, should be carefully harnessed, either shutting a door or opening a window for a great deal of cancer patients.

Aside from the aforementioned issues and back to OVCA, pyroptosis in cancer treatment and cancer patients is another thing to be addressed. Since distinct chemotherapy drugs are of benefit with respect to ovarian cancer *via* stimulation of pyroptosis, along with generation of ROS and decrease of mitochondrial membrane potential, many other precisely targeting pyroptosis medications intended for diverse specific subtypes of ovarian cancer are urgently needed to be developed, as well as more *in vivo* experiments. Besides, the possibility of treating OVCA patients with immunotherapy in conjunction with pyroptosis is worth exploring. Moreover, as mechanisms of pyroptosis in OVCA are still poorly studied, whether unsuspected mechanisms could solve problems related to drug resistance, progression, or recurrence in OVCA patients is yet unknown.

Moreover, there might be a subtle correlation of pyroptosis with ferroptosis and mitochondrial autophagy, which awaits further elucidation. So is it possible to treat OVCA patients with medications that could mediate ovarian cancer cell death through induction of pyroptosis, ferroptosis, necroptosis, and autophagy so as to kill target cells to the greatest degree? Now that a few lncRNAs are reported to regulate pyroptosis in OVCA, chances are that ovarian cancer could be treated at a genetic level. Back to patients themselves, when the pyroptosis progress occurs, a variety of immune components partake including cytotoxic lymphocytes, CAR T cells, IL-1β, and IL-18. Cytotoxic lymphocytes could kill tumor cells by transferring granzymes into target cells. During this process, GSDMB activated by Gzm A or GSDME activated by Gzm B and caspase-3 induces pyroptosis, which probably reinforces the cytotoxicity (125). CAR T cells are supposed to experience a similar course to launch attack, and Gzm B plays a significant role in activating GSDME and caspase-3, as well as inducing pyroptosis. Besides, due to a high affinity between CAR T cells and their ligands, it is more efficient for those cells to induce pyroptosis (126). Moreover, although the cytokines could be properly utilized to assist in fighting against malignancies, for cancer patients, the possibly forthcoming inflammatory cytokine storm under infectious conditions might make things worse. Besides, the newly discovered pyroptosis-related DEGs between OVCA and normal tissues, along with the prognostic and risk models derived from DEGs, might play a critical role in predicting the prognosis of OVCA patients in the future.

Last but not least, there are many FDA-approved drugs in clinical practice that could induce pyroptosis (122). These drugs involve antidiabetes drug metformin, anticancer drugs paclitaxel and doxorubicin, and nutrients anthocyanin and DHA, which show great antitumor activity. In particular, paclitaxel and doxorubicin exhibit enormous potential owing to their dual effects including treating cancers and inducing pyroptosis, but cancer cells could still quickly develop resistance against them, which remains an unsolved but interesting problem. In a study focusing on nasopharyngeal carcinoma (NPC), it was discovered that caspase-1 inhibition and GSDMD knockout could induce a Taxol-resistant phenotype *in vitro* and *in vivo* and that autophagy could negatively regulate the canonical pathway of pyroptosis in NPC cells (127). Additionally, it was also found that the knockdown of USP47, a cysteine protease, could increase doxorubicin-induced pyroptosis in CRC while the ectopic expression of USP47 leads to doxorubicin resistance in CRC cells (128). Thus, we speculate the fact that patients taking particular drugs with dual effects experience drug resistance or tumor relapse might possibly result from the fine regulation of the intricate PCD pathway network. Moreover, although the induction of pyroptosis by these drugs might not directly follow the aforementioned four pathways, the preclinical studies did bring hope to us. Consequently, developing drugs targeting pyroptosis in tumor cells is a promising area. Furthermore, clinical trials regarding pyroptosis do exist, with one focusing on diabetes (129) and the other on leukemia (130). In B cell acute lymphoblastic leukemia patients, B leukemic cell pyroptosis was stimulated through the Gzm B pathway triggered by CAR T cells. However, target cell pyroptosis stimulates macrophages to cause cytokine release syndrome (130), which might be detrimental to patients and become a flaw in pyroptosis, limiting its development. Similarly in patients with type 2 diabetes, alleviation of diabetes *via* inhibiting pyroptosis was observed (129), further confirming the negative inflammatory process during pyroptosis. Therefore, it is inevitable to take the concomitant inflammatory process during pyroptosis into account.

By and large, the continuous exploration centering upon pyroptosis and ovarian cancer provides clinicians with more choices from a genetic level to a chemotherapeutic or an immunotherapeutic level, enriching the thoughts for the treatment of ovarian cancer. Despite some problems to be settled, the significant and promising prospect of pyroptosis is worthy of the wait.

## Author Contributions

TL, MH, and LL had the idea for the article. TL, MH, ML, and LL were the major contributors in the drafting of the work. CQ, LC, TZ, and JQ critically revised the work. All authors contributed to the article and approved the submitted version.

## Conflict of Interest

The authors declare that the research was conducted in the absence of any commercial or financial relationships that could be construed as a potential conflict of interest.

## Publisher’s Note

All claims expressed in this article are solely those of the authors and do not necessarily represent those of their affiliated organizations, or those of the publisher, the editors and the reviewers. Any product that may be evaluated in this article, or claim that may be made by its manufacturer, is not guaranteed or endorsed by the publisher.

## Abbreviations

OVCA, ovarian cancer; Chk, cell cycle checkpoint kinase; PCD, programmed cell death; RIPK3, receptor-interacting protein kinase 3; MLKL, mixed lineage kinase domain-like pseudokinase; GSDM, gasdermin; RD, repressor domain; PFD, pore-forming domain; PRR, pattern-recognition receptor; ASC, apoptosis-associated speck-like protein containing a caspase recruitment domain; AIM2, absent in melanoma 2; NOD, nucleotide-binding oligomerization domain; NLR, NOD-like receptor; PYD, pyrin domain; CARD, caspase recruitment domain; PAMP, pathogen-associated molecular pattern; DAMP, damaged-associated molecular pattern; dsDNA, double-stranded DNA; IL-1β, interleukin-1β; IL-18, interleukin-18; LPS, lipopolysaccharide; P2X7, purinergic receptor P2X, ligand-gated ion channel, 7; NSCLC, non-small cell lung cancer; CRC, colorectal cancer; TNF, tumor necrosis factor; TAK1, TGF-β activated kinase-1; Gzm, granzyme; CAR, chimeric antigen receptor; DEG, differentially expressed gene; lncRNA, long non-coding RNA; GAS5, growth arrest-specific transcript 5; HOTTIP, HOXA transcript at the distal tip; miRNA, microRNA; ASK1, apoptosis signal-regulating kinase1; JNK, c-Jun N-terminal kinase; α-NETA, 2-(alpha-naphthoyl)ethyltrimethylammonium iodide; ROS, reactive oxygen species; MMP, mitochondrial membrane potential; ICI, immune checkpoint inhibitor; NPC, nasopharyngeal carcinoma.

## References

[1] Gaona-Luviano P Medina-Gaona LA Magaña-Pérez K. Epidemiology of Ovarian Cancer. Chin Clin Oncol (2020) 9(4):47. doi: 10.21037/cco-20-34 32648448

[2] Sung H Ferlay J Siegel RL Laversanne M Soerjomataram I Jemal A. Global Cancer Statistics 2020: GLOBOCAN Estimates of Incidence and Mortality Worldwide for 36 Cancers in 185 Countries. CA: Cancer J Clin (2021) 71(3):209–49. doi: 10.3322/caac.21660 33538338

[3] Siegel RL Miller KD. Cancer Statistics, 2021. CA: A Cancer J Clin (2021) 71(1):7–33. doi: 10.3322/caac.21654 33433946

[4] Doubeni CA Doubeni AR Myers AE. Diagnosis and Management of Ovarian Cancer. Am Family Physician (2016) 93(11):937–44. 27281838

[5] Qazi F McGuire WP. The Treatment of Epithelial Ovarian Cancer. CA: Cancer J Clin (1995) 45(2):88–101. doi: 10.3322/canjclin.45.2.88 7889393

[6] Kossaï M Leary A Scoazec JY Genestie C. Ovarian Cancer: A Heterogeneous Disease. Pathobiology J Immunopathol Mol Cell Biol (2018) 85(1-2):41–9. doi: 10.1159/000479006 29020678

[7] Stewart C Ralyea C Lockwood S. Ovarian Cancer: An Integrated Review. Semin Oncol Nurs (2019) 35(2):151–6. doi: 10.1016/j.soncn.2019.02.001 30867104

[8] Barber HR. New Frontiers in Ovarian Cancer Diagnosis and Management. Yale J Biol Med (1991) 64(2):127–41. PMC2589481 1721478

[9] Bond MR Clarke SD Neal FE. Use of Ethoglucid in Treatment of Advanced Malignant Disease. Br Med J (1964) 1(5388):951–3. doi: 10.1136/bmj.1.5388.951 PMC1814150 14107074

[10] Lee JM Minasian L. New Strategies in Ovarian Cancer Treatment. Cancer (2019) 125(Suppl 24):4623–9. doi: 10.1002/cncr.32544 PMC7437367 31967682

[11] Evangelisti G Barra F Moioli M Sala P Stigliani S Gustavino C. Prexasertib: An Investigational Checkpoint Kinase Inhibitor for the Treatment of High-Grade Serous Ovarian Cancer. Expert Opin Investig Drugs (2020) 29(8):779–92. doi: 10.1080/13543784.2020.1783238 32539469

[12] Jorgensen I Rayamajhi M Miao EA. Programmed Cell Death as a Defence Against Infection. Nat Rev Immunol (2017) 17(3):151–64. doi: 10.1038/nri.2016.147 PMC5328506 28138137

[13] Hotchkiss RS Strasser A McDunn JE Swanson PE. Cell Death. N Engl J Med (2009) 361(16):1570–83. doi: 10.1056/NEJMra0901217 PMC3760419 19828534

[14] Tang D Kang R Berghe TV Vandenabeele P. The Molecular Machinery of Regulated Cell Death. Cell Res (2019) 29(5):347–64. doi: 10.1038/s41422-019-0164-5 PMC6796845 30948788

[15] Elmore S. Apoptosis: A Review of Programmed Cell Death. Toxicol Pathol (2007) 35(4):495–516. doi: 10.1080/01926230701320337 17562483 PMC2117903

[16] Fuchs Y Steller H. Live to Die Another Way: Modes of Programmed Cell Death and the Signals Emanating From Dying Cells. Nat Rev Mol Cell Biol (2015) 16(6):329–44. doi: 10.1038/nrm3999 PMC4511109 25991373

[17] Shalini S Dorstyn L Dawar S Kumar S. Old, New and Emerging Functions of Caspases. Cell Death Differ (2015) 22(4):526–39. doi: 10.1038/cdd.2014.216 PMC4356345 25526085

[18] Gudipaty SA Conner CM Rosenblatt J Montell DJ. Unconventional Ways to Live and Die: Cell Death and Survival in Development, Homeostasis, and Disease. Annu Rev Cell Dev Biol (2018) 34:311–32. doi: 10.1146/annurev-cellbio-100616-060748 PMC6791364 30089222

[19] Li L Qiu C Hou M Wang X Huang C Zou J. Ferroptosis in Ovarian Cancer: A Novel Therapeutic Strategy. Front Oncol (2021) 11:665945. doi: 10.3389/fonc.2021.665945 33996593 PMC8117419

[20] Yu ZW Zhang J Li X Wang Y Fu YH Gao XY. A New Research Hot Spot: The Role of NLRP3 Inflammasome Activation, a Key Step in Pyroptosis, in Diabetes and Diabetic Complications. Life Sci (2020) 240:117138. doi: 10.1016/j.lfs.2019.117138 31809715

[21] Zhaolin Z Guohua L Shiyuan W Zuo W. Role of Pyroptosis in Cardiovascular Disease. Cell Prolife (2019) 52(2):e12563. doi: 10.1111/cpr.12563 PMC6496801 30525268

[22] Hu Y Wang B Li S Yang S. Pyroptosis, and Its Role in Central Nervous System Disease. J Mol Biol (2021) 167379. doi: 10.1016/j.jmb.2021.167379 34838808

[23] Hanahan D Weinberg RA. Hallmarks of Cancer: The Next Generation. Cell (2011) 144(5):646. doi: 10.1016/j.cell.2011.02.013 21376230

[24] Kolb R Liu GH Janowski AM Sutterwala FS Zhang W. Inflammasomes in Cancer: A Double-Edged Sword. Protein Cell (2014) 5(1):12–20. doi: 10.1007/s13238-013-0001-4 24474192 PMC3938856

[25] Thi HTH Hong S. Inflammasome as a Therapeutic Target for Cancer Prevention and Treatment. J Cancer Prev (2017) 22(2):62–73. doi: 10.15430/jcp.2017.22.2.62 28698859 PMC5503217

[26] Flores-Romero H Ros U. Pore Formation in Regulated Cell Death. EMBO Journal (2020) 39(23). doi: 10.15252/embj.2020105753 PMC7705454 33124082

[27] Fang Y Tian S Pan Y Li W Wang Q Tang Y. Pyroptosis: A New Frontier in Cancer. Biomed Pharmacother = Biomed Pharmacother (2020) 121:109595. doi: 10.1016/j.biopha.2019.109595 31710896

[28] Tamura M Tanaka S Fujii T Aoki A Komiyama H Ezawa K. Members of a Novel Gene Family, Gsdm, Are Expressed Exclusively in the Epithelium of the Skin and Gastrointestinal Tract in a Highly Tissue-Specific Manner. Genomics (2007) 89(5):618–29. doi: 10.1016/j.ygeno.2007.01.003 17350798

[29] Ding J Wang K Liu W She Y Sun Q Shi J. Pore-Forming Activity and Structural Autoinhibition of the Gasdermin Family. Nature (2016) 535(7610):111–6. doi: 10.1038/nature18590 27281216

[30] Zou J Zheng Y Huang Y Tang D Kang R Chen R. The Versatile Gasdermin Family: Their Function and Roles in Diseases. Front Immunol (2021) 12:751533. doi: 10.3389/fimmu.2021.751533 34858408 PMC8632255

[31] Lin PH Lin HY Kuo CC Yang LT. N-Terminal Functional Domain of Gasdermin A3 Regulates Mitochondrial Homeostasis *via* Mitochondrial Targeting. J Biomed Sci (2015) 22(1):44. doi: 10.1186/s12929-015-0152-0 26100518 PMC4477613

[32] Saeki N Kim DH Usui T Aoyagi K Tatsuta T Aoki K. GASDERMIN, Suppressed Frequently in Gastric Cancer, Is a Target of LMO1 in TGF-Beta-Dependent Apoptotic Signalling. Oncogene (2007) 26(45):6488–98. doi: 10.1038/sj.onc.1210475 17471240

[33] Watabe K Ito A Asada H Endo Y Kobayashi T Nakamoto K. Structure, Expression and Chromosome Mapping of MLZE, a Novel Gene Which Is Preferentially Expressed in Metastatic Melanoma Cells. Jpn J Cancer Res Gann (2001) 92(2):140–51. doi: 10.1111/j.1349-7006.2001.tb01076.x PMC5926699 11223543

[34] Cookson BT Brennan MA. Pro-Inflammatory Programmed Cell Death. Trends Microbiol (2001) 9(3):113–4. doi: 10.1016/s0966-842x(00)01936-3 11303500

[35] Schroder K Tschopp J. The Inflammasomes. Cell (2010) 140(6):821–32. doi: 10.1016/j.cell.2010.01.040 20303873

[36] Aachoui Y Sagulenko V Miao EA Stacey KJ. Inflammasome-Mediated Pyroptotic and Apoptotic Cell Death, and Defense Against Infection. Curr Opin Microbiol (2013) 16(3):319–26. doi: 10.1016/j.mib.2013.04.004 PMC3742712 23707339

[37] Fernandes-Alnemri T Yu JW Datta P Wu J Alnemri ES. AIM2 Activates the Inflammasome and Cell Death in Response to Cytoplasmic DNA. Nature (2009) 458(7237):509–13. doi: 10.1038/nature07710 PMC2862225 19158676

[38] Roberts TL Idris A Dunn JA Kelly GM Burnton CM Hodgson S. HIN-200 Proteins Regulate Caspase Activation in Response to Foreign Cytoplasmic DNA. Science (New York NY) (2009) 323(5917):1057–60. doi: 10.1126/science.1169841 19131592

[39] Guo HT Callaway JB Ting JPY. Inflammasomes: Mechanism of Action, Role in Disease, and Therapeutics. Nat Med (2015) 21(7):677–87. doi: 10.1038/nm.3893 PMC4519035 26121197

[40] Franchi L Eigenbrod T Muñoz-Planillo R Nuñez G. The Inflammasome: A Caspase-1-Activation Platform That Regulates Immune Responses and Disease Pathogenesis. Nat Immunol (2009) 10(3):241–7. doi: 10.1038/ni.1703 PMC2820724 19221555

[41] Fink SL Bergsbaken T Cookson BT. Anthrax Lethal Toxin and Salmonella Elicit the Common Cell Death Pathway of Caspase-1-Dependent Pyroptosis *via* Distinct Mechanisms. Proc Natl Acad Sci USA (2008) 105(11):4312–7. doi: 10.1073/pnas.0707370105 PMC2393760 18337499

[42] Boyden ED Dietrich WF. Nalp1b Controls Mouse Macrophage Susceptibility to Anthrax Lethal Toxin. Nat Genet (2006) 38(2):240–4. doi: 10.1038/ng1724 16429160

[43] Davis BK Wen H Ting JP. The Inflammasome NLRs in Immunity, Inflammation, and Associated Diseases. Annu Rev Immunol (2011) 29:707–35. doi: 10.1146/annurev-immunol-031210-101405 PMC4067317 21219188

[44] Latz E Xiao TS Stutz A. Activation and Regulation of the Inflammasomes. Nat Rev Immunol (2013) 13(6):397–411. doi: 10.1038/nri3452 23702978 PMC3807999

[45] Rathinam VA Fitzgerald KA. Inflammasome Complexes: Emerging Mechanisms and Effector Functions. Cell (2016) 165(4):792–800. doi: 10.1016/j.cell.2016.03.046 27153493 PMC5503689

[46] Sun Q Scott MJ. Caspase-1 as a Multifunctional Inflammatory Mediator: Noncytokine Maturation Roles. J Leukocyte Biol (2016) 100(5):961–7. doi: 10.1189/jlb.3MR0516-224R PMC6608062 27450556

[47] Gurcel L Abrami L Girardin S Tschopp J van der Goot FG. Caspase-1 Activation of Lipid Metabolic Pathways in Response to Bacterial Pore-Forming Toxins Promotes Cell Survival. Cell (2006) 126(6):1135–45. doi: 10.1016/j.cell.2006.07.033 16990137

[48] Zhao Y Yang J Shi J Gong YN Lu Q Xu H. The NLRC4 Inflammasome Receptors for Bacterial Flagellin and Type III Secretion Apparatus. Nature (2011) 477(7366):596–600. doi: 10.1038/nature10510 21918512

[49] Miao EA Mao DP Yudkovsky N Bonneau R Lorang CG Warren SE. Innate Immune Detection of the Type III Secretion Apparatus Through the NLRC4 Inflammasome. Proc Natl Acad Sci USA (2010) 107(7):3076–80. doi: 10.1073/pnas.0913087107 PMC2840275 20133635

[50] Ball DP Taabazuing CY Griswold AR Orth EL Rao SD Kotliar IB. Caspase-1 Interdomain Linker Cleavage Is Required for Pyroptosis. Life Science Alliance (2020) 3(3):e202000664. doi: 10.26508/lsa.202000664 32051255 PMC7025033

[51] Fernandes-Alnemri T Wu J Yu JW Datta P Miller B Jankowski W. The Pyroptosome: A Supramolecular Assembly of ASC Dimers Mediating Inflammatory Cell Death *via* Caspase-1 Activation. Cell Death Differ (2007) 14(9):1590–604. doi: 10.1038/sj.cdd.4402194 PMC3345951 17599095

[52] Broz P Dixit VM. Inflammasomes: Mechanism of Assembly, Regulation and Signalling. Nat Rev Immunol (2016) 16(7):407–20. doi: 10.1038/nri.2016.58 27291964

[53] Shi J Zhao Y Wang K Shi X Wang Y Huang H. Cleavage of GSDMD by Inflammatory Caspases Determines Pyroptotic Cell Death. Nature (2015) 526(7575):660–5. doi: 10.1038/nature15514 26375003

[54] He Y Hara H Nunez G. Mechanism and Regulation of NLRP3 Inflammasome Activation. Trends Biochem Sci (2016) 41(12):1012–21. doi: 10.1016/j.tibs.2016.09.002 PMC5123939 27669650

[55] Guo H Callaway JB Ting JP. Inflammasomes: Mechanism of Action, Role in Disease, and Therapeutics. Nat Med (2015) 21(7):677–87. doi: 10.1038/nm.3893 PMC4519035 26121197

[56] Fink SL Cookson BT. Apoptosis, Pyroptosis, and Necrosis: Mechanistic Description of Dead and Dying Eukaryotic Cells. Infect Immun (2005) 73(4):1907–16. doi: 10.1128/IAI.73.4.1907-1916.2005 PMC1087413 15784530

[57] Nicholson DW Ali A Thornberry NA Vaillancourt JP Ding CK Gallant M. Identification And Inhibition Of the Ice/Ced-3 Protease Necessary for Mammalian Apoptosis. Nature (1995) 376(6535):37–43. doi: 10.1038/376037a0 7596430

[58] Datta D McClendon CL Jacobson MP Wells JA. Substrate and Inhibitor-Induced Dimerization and Cooperativity in Caspase-1 But Not Caspase-3. J Biol Chem (2013) 288(14):9971–81. doi: 10.1074/jbc.M112.426460 PMC3617296 23386603

[59] Boucher D Monteleone M Coll RC. Caspase-1 Self-Cleavage Is an Intrinsic Mechanism to Terminate Inflammasome Activity. J Exp Med (2018) 215(3):827–40. doi: 10.1084/jem.20172222 PMC5839769 29432122

[60] Broz P von Moltke J Jones JW Vance RE Monack DM. Differential Requirement for Caspase-1 Autoproteolysis in Pathogen-Induced Cell Death and Cytokine Processing. Cell Host Microbe (2010) 8(6):471–83. doi: 10.1016/j.chom.2010.11.007 PMC3016200 21147462

[61] Thornberry NA Bull HG Calaycay JR Chapman KT Howard AD Kostura MJ. A Novel Heterodimeric Cysteine Protease Is Required for Interleukin-1 Beta Processing in Monocytes. Nature (1992) 356(6372):768–74. doi: 10.1038/356768a0 1574116

[62] Fantuzzi G Dinarello CA. Interleukin-18 and Interleukin-1 Beta: Two Cytokine Substrates for ICE (Caspase-1). J Clin Immunol (1999) 19(1):1–11. doi: 10.1023/a:1020506300324 10080100

[63] Liu X Zhang Z Ruan J Pan Y Magupalli VG Wu H. Inflammasome-Activated Gasdermin D Causes Pyroptosis by Forming Membrane Pores. Nature (2016) 535(7610):153–8. doi: 10.1038/nature18629 PMC5539988 27383986

[64] Kovacs SB Miao EA. Gasdermins: Effectors of Pyroptosis. Trends Cell Biol (2017) 27(9):673–84. doi: 10.1016/j.tcb.2017.05.005 PMC5565696 28619472

[65] Fink SL Cookson BT. Caspase-1-Dependent Pore Formation During Pyroptosis Leads to Osmotic Lysis of Infected Host Macrophages. Cell Microbiol (2006) 8(11):1812–25. doi: 10.1111/j.1462-5822.2006.00751.x 16824040

[66] Wang WJ Chen D Jiang MZ Xu B Li XW Chu Y. Downregulation of Gasdermin D Promotes Gastric Cancer Proliferation by Regulating Cell Cycle-Related Proteins. J Dig Dis (2018) 19(2):74–83. doi: 10.1111/1751-2980.12576 29314754

[67] Kayagaki N Warming S Lamkanfi M Vande Walle L Louie S Dong J. Non-Canonical Inflammasome Activation Targets Caspase-11. Nature (2011) 479(7371):117–21. doi: 10.1038/nature10558 22002608

[68] Shi J Zhao Y Wang Y Gao W Ding J Li P. Inflammatory Caspases Are Innate Immune Receptors for Intracellular LPS. Nature (2014) 514(7521):187–92. doi: 10.1038/nature13683 25119034

[69] Li P Allen H Banerjee S Franklin S Herzog L Johnston C. Mice Deficient in IL-1 Beta-Converting Enzyme Are Defective in Production of Mature IL-1 Beta and Resistant to Endotoxic Shock. Cell (1995) 80(3):401–11. doi: 10.1016/0092-8674(95)90490-5 7859282

[70] Wang S Miura M Jung YK Zhu H Li E Yuan J. Murine Caspase-11, an ICE-Interacting Protease, Is Essential for the Activation of ICE. Cell (1998) 92(4):501–9. doi: 10.1016/s0092-8674(00)80943-5 9491891

[71] Yang D He Y Munoz-Planillo R Liu Q Nunez G. Caspase-11 Requires the Pannexin-1 Channel and the Purinergic P2X7 Pore to Mediate Pyroptosis and Endotoxic Shock. Immunity (2015) 43(5):923–32. doi: 10.1016/j.immuni.2015.10.009 PMC4795157 26572062

[72] Surprenant A Rassendren F Kawashima E North RA Buell G. The Cytolytic P2Z Receptor for Extracellular ATP Identified as a P2X Receptor (P2X7). Science (New York NY) (1996) 272(5262):735–8. doi: 10.1126/science.272.5262.735 8614837

[73] North RA. Molecular Physiology of P2X Receptors. Physiol Rev (2002) 82(4):1013–67. doi: 10.1152/physrev.00015.2002 12270951

[74] Ruhl S Broz P. Caspase-11 Activates a Canonical NLRP3 Inflammasome by Promoting K(+) Efflux. Eur J Immunol (2015) 45(10):2927–36. doi: 10.1002/eji.201545772 26173909

[75] Hagar JA Powell DA Aachoui Y Ernst RK Miao EA. Cytoplasmic LPS Activates Caspase-11: Implications in TLR4-Independent Endotoxic Shock. Science (New York NY) (2013) 341(6151):1250–3. doi: 10.1126/science.1240988 PMC3931427 24031018

[76] Kayagaki N Wong MT Stowe IB Ramani SR Gonzalez LC Akashi-Takamura S. Noncanonical Inflammasome Activation by Intracellular LPS Independent of TLR4. Science (New York NY) (2013) 341(6151):1246–9. doi: 10.1126/science.1240248 23887873

[77] He WT Wan H Hu L Chen P Wang X Huang Z. Gasdermin D Is an Executor of Pyroptosis and Required for Interleukin-1β Secretion. Cell Res (2015) 25(12):1285–98. doi: 10.1038/cr.2015.139 PMC4670995 26611636

[78] Kayagaki N Stowe IB Lee BL O'Rourke K Anderson K Warming S. Caspase-11 Cleaves Gasdermin D for Non-Canonical Inflammasome Signalling. Nature (2015) 526(7575):666–71. doi: 10.1038/nature15541 26375259

[79] Yokoyama S Nakayama S Xu L Pilon AL Kimura S. Secretoglobin 3A2 Eliminates Human Cancer Cells Through Pyroptosis. Cell Death Discov (2021) 7(1):12. doi: 10.1038/s41420-020-00385-w 33452234 PMC7810848

[80] Soung YH Jeong EG Ahn CH Kim SS Song SY Yoo NJ. Mutational Analysis of Caspase 1, 4, and 5 Genes in Common Human Cancers. Hum Pathol (2008) 39(6):895–900. doi: 10.1016/j.humpath.2007.10.015 18430458

[81] Terlizzi M Colarusso C De Rosa I De Rosa N Somma P Curcio C. Circulating and Tumor-Associated Caspase-4: A Novel Diagnostic and Prognostic Biomarker for Non-Small Cell Lung Cancer. Oncotarget (2018) 9(27):19356–67. doi: 10.18632/oncotarget.25049 PMC5922402 29721208

[82] Terlizzi M Colarusso C De Rosa I Somma P Curcio C Aquino RP. Identification of a Novel Subpopulation of Caspase-4 Positive Non-Small Cell Lung Cancer Patients. J Exp Clin Cancer Res CR (2020) 39(1):242. doi: 10.1186/s13046-020-01754-0 33187551 PMC7664047

[83] Wang Y Gao W Shi X Ding J Liu W He H. Chemotherapy Drugs Induce Pyroptosis Through Caspase-3 Cleavage of a Gasdermin. Nature (2017) 547(7661):99–103. doi: 10.1038/nature22393 28459430

[84] Wang Y Yin B Li D Wang G Han X Sun X. GSDME Mediates Caspase-3-Dependent Pyroptosis in Gastric Cancer. Biochem Biophys Res Commun (2018) 495(1):1418–25. doi: 10.1016/j.bbrc.2017.11.156 29183726

[85] Zhang CC Li CG Wang YF Xu LH He XH Zeng QZ. Chemotherapeutic Paclitaxel and Cisplatin Differentially Induce Pyroptosis in A549 Lung Cancer Cells *via* Caspase-3/GSDME Activation. Apoptosis (2019) 2(3-4):312–25. doi: 10.1007/s10495-019-01515-1 30710195

[86] Yu J Li S Qi J Chen Z Wu Y Guo J. Cleavage of GSDME by Caspase-3 Determines Lobaplatin-Induced Pyroptosis in Colon Cancer Cells. Cell Death Dis (2019) 10(3):193. doi: 10.1038/s41419-019-1441-4 30804337 PMC6389936

[87] Jiang M Qi L Li L Li Y. The Caspase-3/GSDME Signal Pathway as a Switch Between Apoptosis and Pyroptosis in Cancer. Cell Death Discov (2020) 6:112. doi: 10.1038/s41420-020-00349-0 33133646 PMC7595122

[88] Zeng CY Li CG Shu JX Xu LH Ouyang DY Mai FY. ATP Induces Caspase-3/Gasdermin E-Mediated Pyroptosis in NLRP3 Pathway-Blocked Murine Macrophages. Apoptosis (2019) 24(9-10):703–17. doi: 10.1007/s10495-019-01551-x 31175486

[89] Orning P Weng D Starheim K Ratner D. Pathogen Blockade of TAK1 Triggers Caspase-8-Dependent Cleavage of Gasdermin D and Cell Death. Science (New York NY) (2018) 362(6418):1064–9. doi: 10.1126/science.aau2818 PMC6522129 30361383

[90] Sarhan J Liu BC Muendlein HI Li P Nilson R Tang AY. Caspase-8 Induces Cleavage of Gasdermin D to Elicit Pyroptosis During Yersinia Infection. Proc Natl Acad Sci USA (2018) 115(46):E10888–97. doi: 10.1073/pnas.1809548115 PMC6243247 30381458

[91] Schwarzer R Jiao H Wachsmuth L Tresch A Pasparakis M. FADD and Caspase-8 Regulate Gut Homeostasis and Inflammation by Controlling MLKL- and GSDMD-Mediated Death of Intestinal Epithelial Cells. Immunity (2020) 52(6):978–93.e6. doi: 10.1016/j.immuni.2020.04.002 32362323

[92] Fritsch M Günther SD Schwarzer R Albert MC Schorn F Werthenbach JP. Caspase-8 Is the Molecular Switch for Apoptosis, Necroptosis and Pyroptosis. Nature (2019) 575(7784):683–7. doi: 10.1038/s41586-019-1770-6 31748744

[93] Schwarzer R Laurien L Pasparakis M. New Insights Into the Regulation of Apoptosis, Necroptosis, and Pyroptosis by Receptor Interacting Protein Kinase 1 and Caspase-8. Curr Opin Cell Biol (2020) 63:186–93. doi: 10.1016/j.ceb.2020.02.004 32163825

[94] Grossman WJ Revell PA Lu ZH Johnson H Bredemeyer AJ Ley TJ. The Orphan Granzymes of Humans and Mice. Curr Opin Immunol (2003) 15(5):544–52. doi: 10.1016/s0952-7915(03)00099-2 14499263

[95] Voskoboinik I Whisstock JC Trapani JA. Perforin and Granzymes: Function, Dysfunction and Human Pathology. Nat Rev Immunol (2015) 15(6):388–400. doi: 10.1038/nri3839 25998963

[96] Anthony DA Andrews DM Watt SV Trapani JA Smyth MJ. Functional Dissection of the Granzyme Family: Cell Death and Inflammation. Immunol Rev (2010) 235(1):73–92. doi: 10.1111/j.0105-2896.2010.00907.x 20536556

[97] Liu Y Fang Y. Gasdermin E-Mediated Target Cell Pyroptosis by CAR T Cells Triggers Cytokine Release Syndrome. Sci immunol (2020) 5(43):eaax7969. doi: 10.1126/sciimmunol.aax7969 31953257

[98] Zhang Z Zhang Y Xia S Kong Q Li S Liu X. Gasdermin E Suppresses Tumour Growth by Activating Anti-Tumour Immunity. Nature (2020) 579(7799):415–20. doi: 10.1038/s41586-020-2071-9 PMC7123794 32188940

[99] Zhou Z He H. Granzyme A From Cytotoxic Lymphocytes Cleaves GSDMB to Trigger Pyroptosis in Target Cells. Science (New York NY) (2020) 368(6494):eaaz7548. doi: 10.1126/science.aaz7548 32299851

[100] Berkel C Cacan E. Differential Expression and Copy Number Variation of Gasdermin (GSDM) Family Members, Pore-Forming Proteins in Pyroptosis, In Normal and Malignant Serous Ovarian Tissue 233 Spring St. New, York, USA: Springer/PlenumPublishers. Inflammation (2021) 44(6):2203–16. doi: 10.1007/s10753-021-01493-0 34091823

[101] Ye Y Dai Q Qi H. A Novel Defined Pyroptosis-Related Gene Signature for Predicting the Prognosis of Ovarian Cancer. Cell Death Discov (2021) 7(1):71. doi: 10.1038/s41420-021-00451-x 33828074 PMC8026591

[102] Li J Yang C Li Y Chen A Li L You Z. LncRNA GAS5 Suppresses Ovarian Cancer by Inducing Inflammasome Formation. Biosci Rep (2018) 38(2). doi: 10.1042/bsr20171150 PMC5857912 29229673

[103] Tan C Liu W Zheng ZH Wan XG. LncRNA HOTTIP Inhibits Cell Pyroptosis by Targeting miR-148a-3p/AKT2 Axis in Ovarian Cancer. Cell Biol Int (2021) 45(7):1487–97. doi: 10.1002/cbin.11588 33710684

[104] Liang J Zhou J Xu Y Huang X Wang X Huang W. Osthole Inhibits Ovarian Carcinoma Cells Through LC3-Mediated Autophagy and GSDME-Dependent Pyroptosis Except for Apoptosis. Eur J Pharmacol (2020) 874:172990. doi: 10.1016/j.ejphar.2020.172990 32057718

[105] Zhang R Chen J Mao L Guo Y Hao Y Deng Y. Nobiletin Triggers Reactive Oxygen Species-Mediated Pyroptosis Through Regulating Autophagy in Ovarian Cancer Cells. J Agric Food Chem (2020) 68(5):1326–36. doi: 10.1021/acs.jafc.9b07908 31955565

[106] Qiao L Wu X Zhang J Liu L Sui X Zhang R. α-NETA Induces Pyroptosis of Epithelial Ovarian Cancer Cells Through the GSDMD/Caspase-4 Pathway. FASEB J Off Publ Fed Am Soc Exp Biol (2019) 33(11):12760–7. doi: 10.1096/fj.201900483RR 31480859

[107] Zafar S Sarfraz I Rasul A Shah MA Hussain G Zahoor MK. Osthole: A Multifunctional Natural Compound With Potential Anticancer, Antioxidant and Anti-Inflammatory Activities. Mini Rev med Chem (2021) 21(18):2747–63. doi: 10.2174/1389557520666200709175948 32646359

[108] Zhang ZR Leung WN Cheung HY Chan CW. Osthole: A Review on Its Bioactivities, Pharmacological Properties, and Potential as Alternative Medicine. Evidence-Based Complementary Altern Med eCAM (2015) 2015:919616. doi: 10.1155/2015/919616 PMC4515521 26246843

[109] Ashrafizadeh M Zarrabi A Saberifar S Hashemi F Hushmandi K Hashemi F. Nobiletin in Cancer Therapy: How This Plant Derived-Natural Compound Targets Various Oncogene and Onco-Suppressor Pathways. Biomedicines (2020) 8(5). doi: 10.3390/biomedicines8050110 PMC7277899 32380783

[110] Goh JXH Tan LT Goh JK Chan KG Pusparajah P Lee LH. Nobiletin and Derivatives: Functional Compounds From Citrus Fruit Peel for Colon Cancer Chemoprevention. Cancers (2019) 11(6). doi: 10.3390/cancers11060867 PMC6627117 31234411

[111] Wang L Qin X Liang J Ge P. Induction of Pyroptosis: A Promising Strategy for Cancer Treatment. Front Oncol (2021) 11:635774. doi: 10.3389/fonc.2021.635774 33718226 PMC7953901

[112] Ma Y Chen Y Lin C Hu G. Biological Functions and Clinical Significance of the Newly Identified Long Non−Coding RNA RP1−85F18.6 in Colorectal Cancer. Oncol Rep (2018) 40(5):2648–58. doi: 10.3892/or.2018.6694 PMC6151894 30226619

[113] Su F Duan J Zhu J Fu H Zheng X Ge C. Long Non−Coding RNA Nuclear Paraspeckle Assembly Transcript 1 Regulates Ionizing Radiation−Induced Pyroptosis *via* microRNA−448/Gasdermin E in Colorectal Cancer Cells. Int J Oncol (2021) 59(4). doi: 10.3892/ijo.2021.5259 PMC8448542 34476497

[114] Beckwith KS Beckwith MS. Plasma Membrane Damage Causes NLRP3 Activation and Pyroptosis During Mycobacterium Tuberculosis Infection. Nat Commun (2020) 11(1):2270. doi: 10.1038/s41467-020-16143-6 32385301 PMC7210277

[115] Han C Yang Y Guan Q Zhang X Shen H Sheng Y. New Mechanism of Nerve Injury in Alzheimer's Disease: β-Amyloid-Induced Neuronal Pyroptosis. J Cell Mol Med (2020) 24(14):8078–90. doi: 10.1111/jcmm.15439 PMC7348172 32521573

[116] Zhang X Zhang Y Li R Zhu L Fu B Yan T. Salidroside Ameliorates Parkinson's Disease by Inhibiting NLRP3-Dependent Pyroptosis. Aging (2020) 12(10):9405–26. doi: 10.18632/aging.103215 PMC7288953 32432571

[117] Meng Q Li Y Ji T Chao Y Li J Fu Y. Estrogen Prevent Atherosclerosis by Attenuating Endothelial Cell Pyroptosis *via* Activation of Estrogen Receptor α-Mediated Autophagy. J Adv Res (2021) 28:149–64. doi: 10.1016/j.jare.2020.08.010 PMC7753237 33364052

[118] Wang J Deng B Liu Q Huang Y Chen W Li J. Pyroptosis and Ferroptosis Induced by Mixed Lineage Kinase 3 (MLK3) Signaling in Cardiomyocytes Are Essential for Myocardial Fibrosis in Response to Pressure Overload. Cell Death Dis (2020) 11(7):574. doi: 10.1038/s41419-020-02777-3 32710001 PMC7382480

[119] Ding B Sheng J Zheng P Li C Li D Cheng Z. Biodegradable Upconversion Nanoparticles Induce Pyroptosis for Cancer Immunotherapy. Nano Lett (2021) 21(19):8281–9. doi: 10.1021/acs.nanolett.1c02790 34591494

[120] Xiao Y Zhang T Ma X Yang QC Yang LL Yang SC. Microenvironment-Responsive Prodrug-Induced Pyroptosis Boosts Cancer Immunotherapy. Adv Sci (Weinheim Baden-Wurttemberg Germany) (2021) 8(24):e2101840. doi: 10.1002/advs.202101840 PMC8693073 34705343

[121] Zhao P Wang M Chen M Chen Z Peng X Zhou F. Programming Cell Pyroptosis With Biomimetic Nanoparticles for Solid Tumor Immunotherapy. Biomaterials (2020) 254:120142. doi: 10.1016/j.biomaterials.2020.120142 32485591

[122] Tang R Xu J Zhang B Liu J Liang C Hua J. Ferroptosis, Necroptosis, and Pyroptosis in Anticancer Immunity. J Hematol Oncol (2020) 13(1):110. doi: 10.1186/s13045-020-00946-7 32778143 PMC7418434

[123] Janowski AM Kolb R Zhang W Sutterwala FS. Beneficial and Detrimental Roles of NLRs in Carcinogenesis. Front Immunol (2013) 4:370. doi: 10.3389/fimmu.2013.00370 24273542 PMC3824244

[124] Lu X Guo T Zhang X. Pyroptosis in Cancer: Friend or Foe? Cancers (2021) 13(14). doi: 10.3390/cancers13143620 PMC8304688 34298833

[125] Tsuchiya K. Switching From Apoptosis to Pyroptosis: Gasdermin-Elicited Inflammation and Antitumor Immunity. Int J Mol Sci (2021) 22(1). doi: 10.3390/ijms22010426 PMC7794676 33406603

[126] Zhang Z Zhang Y Lieberman J. Lighting a Fire: Can We Harness Pyroptosis to Ignite Antitumor Immunity? Cancer Immunol Res (2021) 9(1):2–7. doi: 10.1158/2326-6066.Cir-20-0525 33397791 PMC7789047

[127] Wang X Li H Li W Xie J Wang F Peng X. The Role of Caspase-1/GSDMD-Mediated Pyroptosis in Taxol-Induced Cell Death and a Taxol-Resistant Phenotype in Nasopharyngeal Carcinoma Regulated by Autophagy. Cell Biol Toxicol (2020) 36(5):437–57. doi: 10.1007/s10565-020-09514-8 31993881

[128] Hou X Xia J Feng Y Cui L Yang Y Yang P. USP47-Mediated Deubiquitination and Stabilization of TCEA3 Attenuates Pyroptosis and Apoptosis of Colorectal Cancer Cells Induced by Chemotherapeutic Doxorubicin. Front Pharmacol (2021) 12:713322. doi: 10.3389/fphar.2021.713322 34630087 PMC8495243

[129] Roshanravan N Alamdari NM Jafarabadi MA Mohammadi A Shabestari BR Nasirzadeh N. Effects of Oral Butyrate and Inulin Supplementation on Inflammation-Induced Pyroptosis Pathway in Type 2 Diabetes: A Randomized, Double-Blind, Placebo-Controlled Trial. Cytokine (2020) 131:155101. doi: 10.1016/j.cyto.2020.155101 32315958

[130] Liu Y Fang Y Chen X Wang Z Liang X Zhang T. Gasdermin E-Mediated Target Cell Pyroptosis by CAR T Cells Triggers Cytokine Release Syndrome. Sci Immunol (2020) 5(43). doi: 10.1126/sciimmunol.aax7969 31953257

